# Pesticide Exposure and Risk of Rheumatoid Arthritis: A Systematic Review and Meta-Analysis

**DOI:** 10.3390/toxics10050207

**Published:** 2022-04-21

**Authors:** Jiraporn Chittrakul, Ratana Sapbamrer, Wachiranun Sirikul

**Affiliations:** Department of Community Medicine, Faculty of Medicine, Chiang Mai University, Chiang Mai 50200, Thailand; jerasooutch@gmail.com (J.C.); wachiranun.sir@gmail.com (W.S.)

**Keywords:** pesticide, insecticide, herbicide, fungicide, rheumatoid arthritis, autoimmune disease

## Abstract

Rheumatoid arthritis (RA) is a disease that affects people all over the world and can be caused by a variety of factors. Exposure to pesticides is one of the risk factors for the development of RA. However, the evidence of exposure to pesticides linked with the development of RA is still controversial. This study aimed to investigate the association between exposure to pesticides and RA by a systematic review of relevant literature and a meta-analysis. Full-text articles published in PubMed, Web of Science, Scopus, and Google Scholar between 1956 and 2021 were reviewed and evaluated. A total of eight studies were eligible for inclusion (two cohort studies, four case-control studies, and two cross-sectional studies). The adjusted odds ratio for pesticide exposure on RA was 1.20 for insecticides (95% CI = 1.12–1.28), 0.98 for herbicides (95% CI = 0.89–1.08), 1.04 for fungicides (95% CI = 0.86–1.27), and 1.15 in for non-specific pesticides (95% CI = 1.09–1.21). There is some evidence to suggest that exposure to insecticides (especially fonofos, carbaryl, and guanidines) contributes to an increased risk of RA. However, the evidence is limited because of a small number of studies. Therefore, further epidemiological studies are needed to substantiate this conclusion.

## 1. Introduction

Rheumatoid arthritis (RA), an autoimmune disease that causes joint inflammation, is a serious public health issue. Between 1980 and 2018, the global prevalence of RA was 460 per 100,000 people [1,2,3,4]. Genetics, smoking, infections, dietary behavior, chemical pollution, and autoimmune illnesses are risk factors for RA [3,5,6]. It has been reported that exposure to pesticides causes inflammation within the immune system that is directly toxic to that system, leading to chronic disease including RA [7]. It has also been found that RA affects not only the physical but also the socio-economic effects of the patient, including medical costs and the loss of disability income in work [8,9]. The focus of this study is to clearly identify the modifiable factors that cause RA, facilitating early intervention in those at risk of RA.

Despite increased awareness of the dangers associated with pesticide poisoning, it is still a major worldwide public health issue [10]. Pesticides are chemicals used in agriculture, gardening operations, and some house-cleaning products. Pesticides are usually classified by their pest target, for example, insecticides, herbicides, and fungicides [11]. Pesticides can enter the body by contact with skin, ingestion, and inhalation [12]. Doses and duration of exposure to pesticides are crucial factors in both acute and chronic health effects. Acute poisoning is caused by a single exposure to a high dosage of a pesticide, while chronic conditions are adverse health effects resulting from long-term exposure to pesticides [12,13]. Long term exposure to pesticides can disrupt organ functions such as those in the nervous system, endocrine system, respiratory system, reproductive system, kidney system, cardiovascular system, and immune system, resulting in conditions such as cancer, Parkinson’s disease, Alzheimer’s disease, multiple sclerosis, diabetes, coronary heart disease, chronic kidney disease, respiratory diseases, autoimmune diseases, and systemic lupus erythematosus [6,14,15,16]. RA is a major immune system disease, according to early studies suggesting that pesticides damage humans’ immune system. Moreover, some studies have shown that occupational exposure to pesticides was linked with the development of RA [6], whereas other studies failed to confirm such an association [17,18]. As a consequence, the evidence available with regard to the link between pesticides and RA was inconsistent. As this is a contentious, rapidly changing field, the evidence needs to be continuously, systematically reviewed to update the state of knowledge. The aim of this study is to carry out a systematic review and meta-analysis of the existing literature specifically on the effects of pesticides on RA.

## 2. Materials and Methods

This review was performed in accordance with the PRISMA (Preferred Reporting Items for Systematic Reviews and Meta-Analyses) guidelines, and we have registered PROSPERO, registration number 4202299598.

### 2.1. Searching Strategy

This study aimed to review scientific evidence and carry out a meta-analysis of exposure to pesticides contributing to RA. The study was carried out in accordance with the Preferred Reporting Items for Systematic Reviews and Meta-Analysis (PRISMA). PubMed, Web of Science, Scopus, and Google Scholar were searched for full-text articles using the following keywords: “rheumatoid arthritis” OR “autoimmune disease” plus “pesticide” OR “herbicide” OR “insecticide” OR “fungicide”. The study was registered under PROSPERO (registration number: CRD42022299598, 20 January 2022). The final search was completed on 14 February 2022. The search process was performed by all authors.

### 2.2. Inclusion Criteria

The inclusion criteria were as follows: (1) original article; (2) full-text article; (3) published between 1956 and 2021; (4) written in the English language; (5) assessed RA by a diagnosis of physicians or self-reported; (6) assessed the association between pesticide exposure and rheumatoid arthritis; and (7) data analysis was by regression analysis, or discriminant analysis for adjustment of confounding variables. The studies that were review articles, had irrelevant information, or were without variables of interest were excluded from the study.

**Figure 1:**
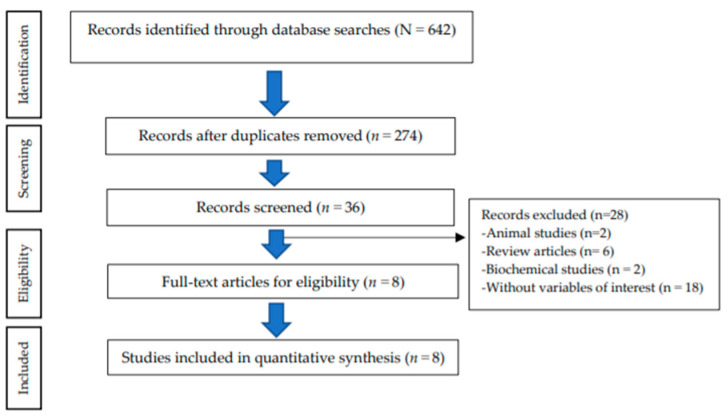
Flow chart of the study selection process (PRISMA).

The study selection process resulted in the following: 642 records identified through the databases; 274 records remained after duplicate removal; 36 articles remained after screening for full-text articles; finally resulting in 8 eligible articles for inclusion in the quantitative synthesis. A total of 28 articles were excluded because of being animal studies (*n* = 2), review articles (*n* = 6), biochemical studies (*n* = 2), and without variables of interest (*n* = 18) (Figure 1). 

### 2.3. Data Extraction and Quality Assessment

The data from eligible articles were independently extracted by two investigators. The extracted data were as follows: the name of the first author, publication year, country, study design, number of the population, age, gender, name of the chemical, adjusted odds ratio (aOR), 95% confidence intervals (95% CI), and confounding variables.

The quality of the eligible articles was assessed using National Heart, Lung, and Blood Institute (NHLBI) Guidelines for reporting observational cohort, cross-sectional, and case-control studies [19]. The NHBLI checklist consisted of 14 items for reporting observational cohort and cross-sectional studies and 12 items for reporting case-control studies. The quality of the eligible articles was independently assessed by three reviewers (Tables S1 and S2).

### 2.4. Data Analysis

The eight studies selected were divided into four groups on the basis of the different types of pesticides, insecticides, herbicides, fungicides, and non-specific pesticides. A fixed-effect model with the Mantel–Haenszel method was used for analysis. The random-effect model of the DerSiomonian and Laird method was also used. The heterogeneity of selected studies was confirmed using the Cochran Q and I^2^ tests. The heterogeneity was categorized into three criteria: low heterogeneity (I^2^ < 25%); moderate heterogeneity (I^2^ 25–50%); substantial heterogeneity (I^2^ > 50%). Funnel plots were tested to identify the publication bias of the selected studies. OR was plotted in the horizontal axis of the funnel plot, and standard error on the vertical axis. Two-tailed statistical tests at *p*-value < 0.05 were used. The data were analyzed using the STATA software package (Stata Corp. 2019. Stata Statistical Software: Release 16. College Station, TX, USA: Stata Corp LLC.).

## 3. Results

### 3.1. Association between Exposure to Insecticides and RA Development

Five studies were eligible for inclusion in the quantitative synthesis [6,17,18,20,21]: one study was a cohort study, two were case-control studies, and two were cross-sectional studies. Three studies were conducted in the USA (*n* = 3), with the others being conducted in Norway (*n* = 1) and Greece (*n* = 1). Of the five studies, two studies found an association between exposure to insecticides and RA, but three studies found no association. The study by Meyer et al. [6] found an association of increased risk of RA with exposure to fonofos (aOR = 1.7, 95% CI = 1.22–2.37) and carbaryl (aOR = 1.51, 95% CI = 1.03–2.23). The study by Koureas et al. [21] also found an association of increased risk of RA with exposure to organophosphates (aOR = 6.47, 95% CI = 1.00–45.43) and guanidines (aOR = 16.18, 95% CI = 1.58–165.97) (Table 1).

**Figure 2:**
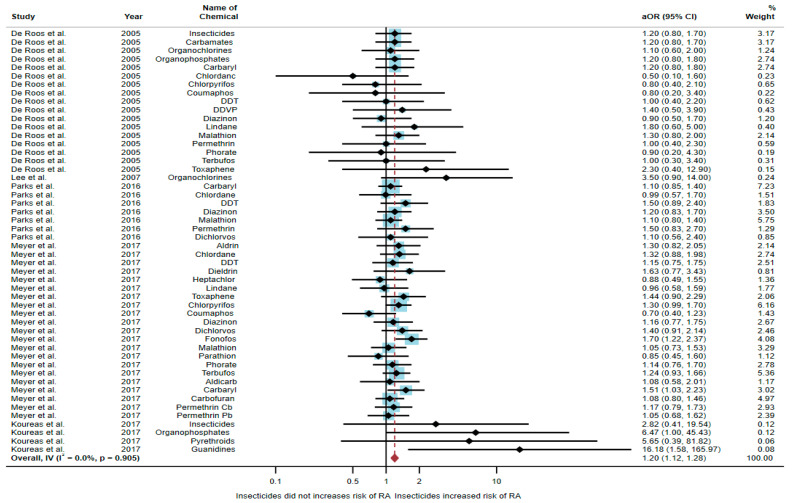
The association between exposure to insecticides and RA development [6,17,18,20,21]. aOR, adjusted odds ratio; 95% CI, 95% confidence interval.

A total of 52,896 participants were included in the meta-analysis, and exposure to insecticides was found to be significantly associated with an increased risk of RA (aOR = 1.20, 95% CI = 1.12–1.28, *p*-value = 0.905 for heterogeneity I^2^ = 0%) (Figure 2).

### 3.2. Association between Exposure to Herbicides and RA Development

Four studies were eligible for inclusion in the quantitative synthesis [6,17,20,21]: one study was a cohort study, two were case-control studies, and one was a cross-sectional study. Three studies were conducted in the USA (*n* = 3), and one was conducted in Greece (*n* = 1). Of the four studies, only one study found an association between exposure to herbicides and RA, but three studies found no association. The study by Meyer et al. [6] found an association of increased risk of RA with exposure to chlorimuron ethyl (aOR = 1.45, 95% CI = 1.01–2.07) and EPTC (aOR = 0.62, 95% CI = 0.4–0.96). However, the study by De Roos et al. [17] found a negative association between RA and exposure to phenoxyacetic acids (aOR = 0.5, 95% CI = 0.3–0.9) and 2,4-D (aOR = 0.5, 95% CI = 0.3–0.9) (Table 2).

**Figure 3:**
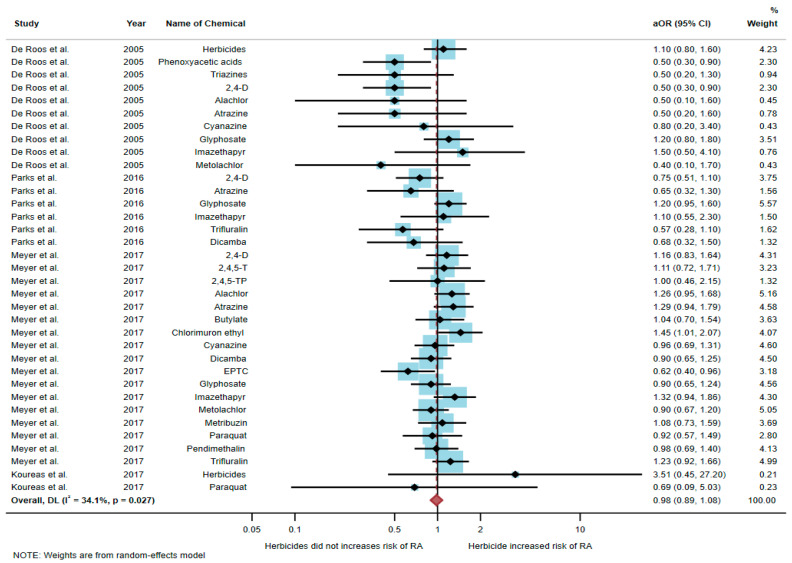
The association between exposure to herbicides and RA development [6,17,20,21]. aOR, adjusted odds ratio; 95% CI, 95% confidence interval.

A total of 51,175 participants were included in the meta-analysis, and the results found that exposure to herbicides was not significantly associated with an increased risk of RA (aOR = 0.98, 95% CI = 0.89–1.08, *p*-value = 0.027 for heterogeneity I^2^ = 34.1%) (Figure 3).

### 3.3. Association between Exposure to Fungicides and RA Development

Four studies were eligible for inclusion in the quantitative synthesis [6,17,20,21]: one study was a cohort study, two were case-control studies, and one was a cross-sectional study. Three studies were conducted in the USA (*n* = 3), and one was conducted in Greece (*n* = 1). Of the four studies, only one study found an association between exposure to fungicides and RA, but three studies found no association. The study by Parks et al. [20] found an association of increased risk of RA with exposure to maneb/mancozeb (aOR = 2.0, 95% CI = 1.1–3.9) (Table 3).

**Figure 4:**
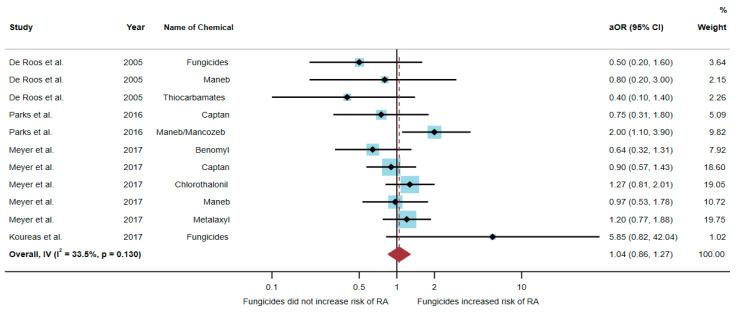
The association between exposure to fungicides and RA development [6,17,20,21]. aOR, adjusted odds ratio; 95% CI, 95% confidence interval.

A total of 51,175 participants were included in the analysis, and the results found that exposure to fungicides was not significantly associated with an increased risk of RA (aOR = 1.04, 95% CI = 0.86–1.27, *p*-value = 0.130 for heterogeneity I^2^ = 33.5%) (Figure 4).

### 3.4. Association between Exposure to Non-Specific Pesticides and RA Development

Four studies were eligible for inclusion in the quantitative synthesis [20,22,23,24]: two studies were cohort studies, and two were case-control studies. Three studies were conducted in the USA (*n* = 3), and one was conducted in Sweden (*n* = 1).

Out of the four articles, two studies found an association between exposure to non-specific pesticides and RA, but two studies found no association. The study by Parks et al. [24] found an association with non-specific pesticides (aOR = 1.8, 95% CI = 1.1–2.9). Similarly, the study by Gold et al. [23] also found an association with non-specific pesticides (aOR = 1.14, 95% CI = 1.08–1.2) (Table 4).

**Figure 5:**
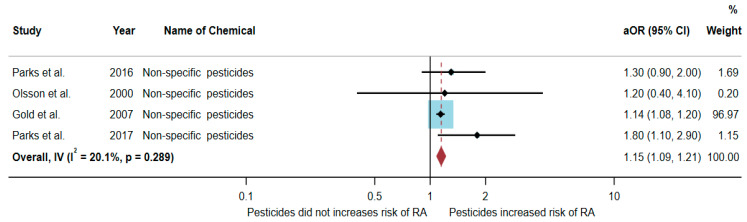
The association between exposure to non-specific pesticides and RA development [20,22,23,24]. aOR, adjusted odds ratio; 95% CI, 95% confidence interval.

A total of 369,896 participants were included in the meta-analysis, and the results showed that exposure to non-specific pesticides was significantly associated with an increased risk of RA (aOR = 1.15, 95% CI = 1.09–1.21, *p*-value = 0.289 for heterogeneity I^2^ = 20.1%) (Figure 5).

### 3.5. Funnel Plots

**Figure 6:**
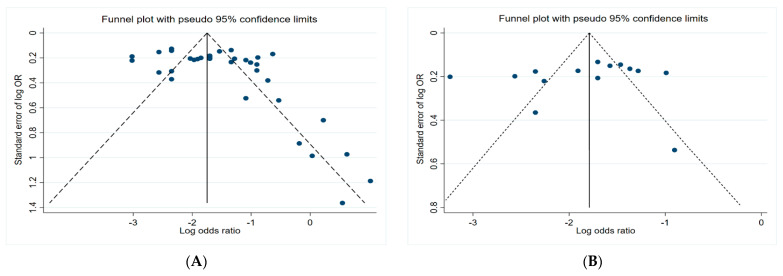

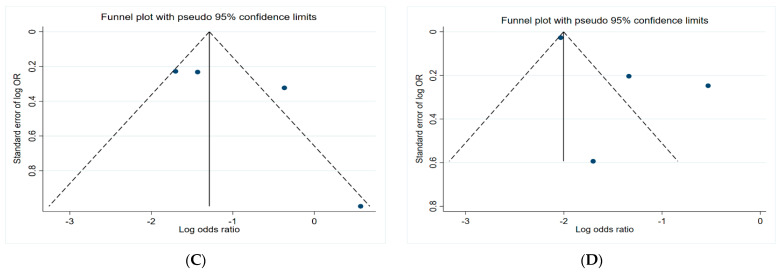
Funnel plots. (**A**) Insecticides; (**B**) herbicides; (**C**) fungicides; (**D**) non-specific pesticides.

Figure 6 presents the funnel plots of the subgroups of the studies, including insecticides, herbicides, fungicides, and non-specific pesticides. The results indicate that all funnel plots are asymmetrical, which may be a result of several factors, for example, the magnitude of the effect may vary with the study size and location bias.

## 4. Discussion

The studies currently available provided evidence that exposure to insecticides contributed to increased risk of RA (aOR = 1.20, 95% CI = 1.12–1.28). In addition, the specific insecticides that found a significant association were fonofos, cabaryl, and guanidines. Fonophos belongs to the organophosphate group, carbaryl is a carbamate, and guanidine a neonicotinoid. Importantly, the studies that found the association of exposure to insecticides with an increased risk of RA was found in insecticide applicators [6,21]. Therefore, this evidence provides considerable information that occupational exposure to insecticides is a possible risk factor for the development of RA. 

RA is an illness of the immune system that causes joint inflammation [25]. Genetics and environmental factors are the two main divisions of risk factors for RA. Environmental risk factors are intriguing because they have the potential to affect a variety of physiological systems, including the immune system [26]. Pesticides have been found to be a major environmental risk factor. Several studies have revealed that pesticide exposure can harm the immune system and contribute to the onset of RA [6,20,21,23,26].

Direct immunotoxicity is the main mechanism associated with the impact of pesticide poisoning on the immune system [27]. Inhibition of acetylcholinesterase (AChE) by organophosphates and carbamates can result in inhibition of cholinergic signaling in lymphocytes. Exposure to high levels of insecticides can lead to accumulation of the neurotransmitter acetylcholine and overstimulation of cholinergic receptors. As a result, interleukin-2 (IL-2) is produced by T cells and B cells, resulting in stimulation of the inflammatory response in macrophages, which increases the risk of inflammatory disease [7,27,28]. However, exposure to low levels of insecticides for long periods may lead to a reduction in cholinergic receptors, which could lead to chronic disease or cancer [27].

T cells are lymphocytes that play an important role in immunity. Previously available studies have shown that insecticides had a negative effect on the viability and function of T cells by inducing apoptosis in different ways [29]. A previous study found that exposure to carbamates can induce apoptosis in human Jurkat T cells [29]. An investigation in an animal model also suggested that the intrinsic apoptotic pathway was stimulated due to the increase in the levels of caspase 3 and cytochrome C released from the mitochondria [30]. Carbamates also affect lymphocyte-specific kinases, resulting in an inhibitory effect on T cell and IL-2 production [29,31]. Several studies also suggested that chlorpyrifos and dimethyl 2,2-dichlorovinyl phosphate can inhibit cytotoxic T lymphocyte activity and decrease the T cell population [29,32,33]. With regard to B cells, pesticides can also inhibit B cell proliferation, leading to lower levels of B cells and reduced antibody production [29]. Some environmental chemicals can trigger an immunological response specific to antigens, which can cause polyclonal B cells to produce antibodies against themselves in some individuals, an autoimmune response [34]. Insecticides can also inhibit the development of M1 macrophages and increase M2 macrophage polarization, which leads to immunotoxicity in humans [27,28,29,35]. In addition, inhibition of the transcription levels of pro-inflammatory cytokines, which are related to oxidative stress, might be caused by insecticides. This results in increasing ROS and DNA damage, as well as the induction of apoptosis [36,37]. 

Although the studies currently available provided evidence that exposure to insecticides contributes to an increased risk of RA, there were some limitations in the study reviews. Firstly, some studies assessed the association between exposure to pesticides and RA by using cross-sectional studies; therefore, these studies were unable to describe the relationships between cause and effect. Secondly, most studies were conducted in the USA; therefore, the evidence cannot be directly generalized to other populations. Thirdly, the number of eligible studies was rather small; therefore, the interpretation of the evidence should be more careful. Fourthly, the eligible studies included for meta-analysis were both occupational and environmental exposure; therefore, the interpretation of the evidence should be concerned. Fifthly, most studies assessed the exposure to pesticides by using interviews or self-reporting procedures that may not quantitatively indicate the level of exposure and therefore may not be able to be directly correlated with the development of RA. Sixthly, exposure to multiple pesticides might result in the development of RA. Previous studies stated that co-exposure to pesticides affects toxicity in humans and may lead to chronic illness [38,39]. Therefore, further studies should be concerned on this point. Finally, there were several confounding factors contributing to RA. Therefore, in future studies, the confounding factors need to be considered, including age, race, genetics, income status, body mass index, resident area, occupation, education level, cigarette smoking and alcohol consumption, and exposure to other environmental chemicals.

## 5. Conclusions

There is some evidence to suggest that exposure to insecticides (especially fonofos, carbaryl, and guanidines) contributes to an increased risk of RA, while exposure to herbicides and fungicides has no impact on the development of RA. However, the evidence is limited because of a small number of studies. Therefore, further studies regarding the effect of pesticides on the development of RA need to be warranted. A continuous approach needs to be adopted to systematic review and meta-analysis of the evidence to ensure current updating of the knowledge pertinent to the effects of pesticides on RA development.

## Figures and Tables

**Table 1:** The association between exposure to insecticides and RA development.

Authors (Years)/Country	Study Design	Gender	Sample Size	Name of Chemicals	aOR (95% CI)	Confounding Variables
De Roos et al. (2005)/USA [17]	Case–control	Female	810	Insecticides Carbamates Organochlorines Organophosphates Carbaryl Chlordane Chlorpyrifos Coumaphos DDT DDVP Diazinon Lindane Malathion Permethrin Phorate Terbufos Toxaphene	1.2 (0.8–1.7) 1.2 (0.8–1.7) 1.1 (0.6–2.0) 1.2 (0.8–1.8) 1.2 (0.8–1.8) 0.5 (0.1–1.6) 0.8 (0.4–2.1) 0.8 (0.2–3.4) 1.0 (0.4–2.2) 1.4 (0.5–3.9) 0.9 (0.5–1.7) 1.8 (0.6–5.0) 1.3 (0.8–2.0) 1.0 (0.4–2.3) 0.9 (0.2–4.3) 1.0 (0.3–3.4) 2.3 (0.4–12.9)	Birth date, and state
Lee et al. (2007)/ Norway [18]	Cross-sectional	Both genders	1721	Organochlorines	3.5 (0.9–14.0)	Age, race, income status, BMI, and cigarette smoking
Parks et al. (2016)/USA [20]	Cohort	Female	23,841	Carbaryl Chlordane DDT Diazinon Malathion Permethrin Dichlorvos	1.1 (0.85–1.4) 0.99 (0.57–1.7) 1.5 (0.89–2.4) 1.2 (0.83–1.7) 1.1 (0.80–1.4) 1.5 (0.83–2.7) 1.1 (0.56–2.4)	Age, state, and smoking pack-years
Meyer et al. (2017)/USA [6]	Case–control	Male	26,354	Aldrin Chlordane DDT Dieldrin Heptachlor Lindane Toxaphene Chlorpyrifos Coumaphos Diazinon Dichlorvos Fonofos Malathion Parathion Phorate Terbufos Aldicarb Carbaryl Carbofuran Permethrin ^a^ Permethrin ^b^	1.30 (0.82–2.05) 1.32 (0.88–1.98) 115 (0.75–1.75) 1.63 (0.77–3.43) 0.88 (0.49–1.55) 0.96 (0.58–1.59) 1.44 (0.90–2.29) 1.30 (0.99–1.70) 0.70 (0.40–1.23) 1.16 (0.77–1.75) 1.40 (0.91–2.14) 1.70 (1.22–2.37) 1.05 (0.73–1.53) 0.85 (0.45–1.60) 1.14 (0.76–1.70) 1.24 (0.93–1.66) 1.08 (0.58–2.01) 1.51 (1.03–2.23) 1.08 (0.80–1.46) 1.17 (0.79–1.73) 1.05 (0.68–1.62)	Age, state of enrollment, pack-years smoking, and education level
Koureas et al. (2017)/Greece [21]	Cross- sectional	Male	170	Insecticides Organophosphates Pyrethroids Guanidines	2.82 (0.41–19.54) 6.47 (1.00–45.43) 5.65 (0.39–81.82) 16.18 (1.58–165.97)	Age, smoker, alcohol consumption, and use of a tractor on a farm

^a^ Used on crops; ^b^ used on poultry and livestock; aOR, adjusted odds ratio; 95% CI, 95% confidence interval; DDT, dichlorodiphenyltrichloroethane; DDVP, 2,2-dichlorovinyl dimethyl phosphate.

**Table 2:** The association between exposure to herbicides and RA development.

Authors (Years)/Country	Study Design	Gender	Sample Size	Name of Chemicals	aOR (95% CI)	Confounding Variables
De Roos et al. (2005)/USA [17]	Case–control	Female	810	Herbicides Phenoxyacetic acids Triazines 2,4-D Alachlor Atrazine Cyanazine Glyphosate Imazethapyr Metolachlor	1.1 (0.8–1.6) 0.5 (0.3–0.9) 0.5 (0.2–1.3) 0.5 (0.3–0.9) 0.5 (0.1–1.6) 0.5 (0.2–1.6) 0.8 (0.2–3.4) 1.2 (0.8–1.8) 1.5 (0.5–4.1) 0.4 (0.1–1.7)	Birth date and state
Parks et al. (2016)/USA [20]	Cohort	Female	23,841	2,4-D Atrazine Glyphosate Imazethapyr Trifluralin Dicamba	0.75 (0.51–1.1) 0.65 (0.32–1.3) 1.2 (0.95–1.6) 1.1 (0.55–2.3) 0.57 (0.28–1.1) 0.68 (0.32–1.5)	Age, state, and pack-years smoking
Meyer et al. (2017)/USA [6]	Case–control	Male	26,354	2,4-D 2,4,5-T 2,4,5-TP Alachlor Atrazine Butylate Chlorimuron ethyl Cyanazine Dicamba EPTC Glyphosate Imazethapyr Metolachlor Metribuzin Paraquat Pendimethalin Trifluralin	1.16 (0.83–1.64) 1.11 (0.72–1.71) 1.00 (0.46–2.15) 1.26 (0.95–1.68) 1.29 (0.94–1.79) 1.04 (0.70–1.54) 1.45 (1.01–2.07) 0.96 (0.69–1.31) 0.90 (0.65–1.25) 0.62 (0.40–0.96) 0.90 (0.65–1.24) 1.32 (0.94–1.86) 0.90 (0.67–1.20) 1.08 (0.73–1.59) 0.92 (0.57–1.49) 0.98 (0.69–1.40) 1.23 (0.92–1.66)	Age, state of enrollment, pack-years smoking, and education level
Koureas et al. (2017)/Greece [21]	Cross- sectional	Male	170	Herbicides Paraquat	3.51 (0.45–27.20) 0.69 (0.09–5.03)	Age, smoker, alcohol consumption, and use of a tractor on a farm

aOR, adjusted odds ratio; 95% CI, 95% confidence interval; 2,4-D, 2,4-dichlorophenoxyacetic acid; 2,4,5-T, 2,4,5-trichlorophenoxyacetic acid; 2,4,5-TP, 2,4,5-trichlorophenoxy propionic acid; EPTC, S-ethyl-N, N-dipropylthiocarbamate.

**Table 3:** The association between exposure to fungicides and RA development.

Authors (Years)/Country	Study Design	Gender	Sample Size	Name of Chemicals	aOR (95% CI)	Confounding Variables
De Roos et al. (2005)/USA [17]	Case–control	Female	810	Fungicides Maneb Thiocarbamates	0.5 (0.2–1.6) 0.8 (0.2–3.0) 0.4 (0.1–1.4)	Birth date and state
Parks et al. (2016)/USA [20]	Cohort	Female	23,841	Captan Maneb/Mancozeb	0.75 (0.31–1.8) 2.0 (1.1–3.9)	Age, state, and pack-years smoking
Meyer et al. (2017)/USA [6]	Case–control	Male	26,354	Benomyl Captan Chlorothalonil Maneb Metalaxyl	0.64 (0.32–1.31) 0.90 (0.57–1.43) 1.27 (0.81–2.01) 0.97 (0.53–1.78) 1.20 (0.77–1.88)	Age, state of enrollment, pack-years smoking, and education level
Koureas et al. (2017)/Greece [21]	Cross-sectional	Male	170	Fungicides	5.85 (0.82–42.04)	Age, smoker, alcohol consumption, and use of a tractor on a farm

aOR, adjusted odds ratio; 95% CI, 95% confidence interval.

**Table 4:** The association between exposure to non-specific pesticides and RA development.

Authors (Years)/ Country	Study Design	Gender	Sample Size	Adjusted OR (95% CI)	Confounding Variables
Parks et al. (2016)/USA [20]	Cohort	Female	23,841	1.3 (0.9–2.0)	Age, state, and pack-years smoking
Olsson et al. (2000)/Sweden [22]	Case–control	Male	350	1.2 (0.4–4.1)	Age, smoking, and occupation
Gold et al. (2007)/USA [23]	Case–control	Both genders	296,362	1.14 (1.08–1.20)	Age, sex, race, region, and socioeconomic status
Parks et al. (2017)/USA [24]	Cohort	Female	49,343	1.8 (1.1–2.9)	Age, race, education level, packyears of smoking, and childhood socioeconomic status

aOR, adjusted odds ratio; 95% CI, 95% confidence interval.

## Data Availability

Not applicable.

## Supplementary Materials

The following supporting information can be downloaded at https://www.mdpi.com/article/10.3390/toxics10050207/s1, Table S1. Quality assessment of selected articles in accordance with the National Heart, Lung, and Blood Institute (NHLBI) Guidelines for reporting observational cohort and cross-sectional studies; Table S2. Quality assessment of selected articles in accordance with the National Heart, Lung, and Blood Institute (NHLBI) Guidelines for reporting case-control studies.
