# Supplementary material for: Pesticide Exposure and Risk of Rheumatoid Arthritis: A Systematic Review and Meta-Analysis
Source: Toxics. 2022 Apr 21;10(5):207. doi: 10.3390/toxics10050207 (PMC9143500; doi:10.3390/toxics10050207)
Supplement: Supplementary file 1 [file toxics-10-00207-s001.zip › toxics-1635248-supplementary.pdf]

toxics MDPI

Supplementary Materials: Pesticide Exposure and Risk of Rheumatoid Arthritis: A Systematic Review and Meta-Analysis

Jiraporn Chittrakul, Ratana Sapbamrer * and Wachiranun Sirikul

Table S1. Quality assessment of selected articles in accordance with the National Heart, Lung, and Blood Institute (NHLBI) Guidelines for reporting observational cohort and cross-sectional studies

Study	Criteria													
	1	2	3	4	5	6	7	8	9	10	11	12	13	14
**Cohort studies**														
Parks et al. (2016)	yes	yes	yes	yes	NR	yes	yes	yes	yes	yes	yes	yes	yes	yes
Parks et al. (2017)	yes	yes	yes	yes	NR	yes	yes	yes	yes	yes	yes	yes	yes	yes
**Cross-sectional studies**														
Lee et al. (2007)	yes	yes	yes	yes	NR	yes	yes	yes	yes	NA	yes	yes	CD	yes
Koureas et al.(2017)	yes	yes	yes	yes	NR	yes	yes	yes	yes	NA	yes	no	CD	yes

CD, cannot determine; NA, not applicable; NR, not reported

Criteria1. Was the research question or objective in this paper clearly stated?
Criteria 2. Was the study population clearly specified and defined?
Criteria 3. Was the participation rate of eligible persons at least 50%?
Criteria 4. Were all the subjects selected or recruited from the same or similar populations (including the same time period)? Were inclusion and exclusion criteria for being in the study prespecified and applied uniformly to all participants?
Criteria 5. Was a sample size justification, power description, or variance and effect estimates provided?
Criteria 6. For the analyses in this paper, were the exposure(s) of interest measured prior to the outcome(s) being measured?
Criteria 7. Was the timeframe sufficient so that one could reasonably expect to see an association between exposure and outcome if it existed?
Criteria 8. For exposures that can vary in amount or level, did the study examine different levels of the exposure as related to the outcome (e.g., categories of exposure, or exposure measured as continuous variable)?
Criteria 9. Were the exposure measures (independent variables) clearly defined, valid, reliable, and implemented consistently across all study participants?
Criteria 10. Was the exposure(s) assessed more than once over time?
Criteria 11. Were the outcome measures (dependent variables) clearly defined, valid, reliable, and implemented consistently across all study participants?
Criteria 12. Were the outcome assessors blinded to the exposure status of participants?
Criteria 13. Was loss to follow-up after baseline 20% or less?
Criteria 14. Were key potential confounding variables measured and adjusted statistically for their impact on the relationship between exposure(s) and outcome(s)?

Table S2. Quality assessment of selected articles in accordance with the National Heart, Lung, and Blood Institute (NHLBI) Guidelines for reporting case-control studies.

**Study**	**Criteria**											
	**1**	**2**	**3**	**4**	**5**	**6**	**7**	**8**	**9**	**10**	**11**	**12**
De Roos et al. (2005)	yes	yes	yes	yes	yes	yes	yes	yes	yes	yes	yes	yes
Meyer et al. (2017)	yes	yes	yes	yes	yes	yes	yes	yes	yes	yes	yes	yes
Gold et al. (2007)	yes	yes	yes	yes	yes	yes	yes	yes	yes	yes	no	yes

CD, cannot determine; NA, not applicable; NR, not reported

Criteria1. Was the research question or objective in this paper clearly stated and appropriate?

Criteria 2. Was the study population clearly specified and defined?

Criteria 3. Did the authors include a sample size justification?

Criteria 4. Were controls selected or recruited from the same or similar population that gave rise to the cases (including the same timeframe)?

Criteria 5. Were the definitions, inclusion and exclusion criteria, algorithms or processes used to identify or select cases and controls valid, reliable, and implemented consistently across all study participants?

Criteria 6. Were the cases clearly defined and differentiated from controls?

Criteria 7. If less than 100 percent of eligible cases and/or controls were selected for the study, were the cases and/or controls randomly selected from those eligible?

Criteria 8. Was there use of concurrent controls?

Criteria 9. Were the investigators able to confirm that the exposure/risk occurred prior to the development of the condition or event that defined a participant as a case?

Criteria 10. Were the measures of exposure/risk clearly defined, valid, reliable, and implemented consistently (including the same time period) across all study participants?

Criteria 11. Were the assessors of exposure/risk blinded to the case or control status of participants?

Criteria 12. Were key potential confounding variables measured and adjusted statistically in the analyses? If matching was used, did the investigators account for matching during study analysis?
